# Inhibition of Macrophage Migration Inhibitory Factor Protects against Inflammation and Matrix Deposition in Kidney Tissues after Injury

**DOI:** 10.1155/2016/2174682

**Published:** 2016-05-23

**Authors:** Hong Lu, Yongyu Bai, Lianfeng Wu, Weilong Hong, Yong Liang, Bicheng Chen, Yongheng Bai

**Affiliations:** ^1^Department of Laboratory Medicine, The First Affiliated Hospital of Wenzhou Medical University, Wenzhou 325000, China; ^2^Key Laboratory of Surgery, The First Affiliated Hospital of Wenzhou Medical University, Wenzhou 325000, China

## Abstract

*Background*. Macrophage migration inhibitory factor (MIF) is an important immunoregulatory cytokine involved in inflammation, which may be one important reason resulting in matrix deposition in renal tissues after injury. However, the underlying mechanisms have not yet been elucidated.* Methods and Results*. We uncovered a crucial role of MIF in inflammation and collagen deposition* in vivo* and* in vitro*. In rats, ureteral obstruction induced tubular injury, matrix accumulation, and inflammatory cell infiltration. Additionally, enhanced MIF levels in the obstructed kidneys were closely related to the increasing numbers of CD68-positive macrophages. These obstruction-induced injuries can be relieved by recanalization, consequently resulting in downregulated expression of MIF and its receptor CD74. Similarly, ischemia reperfusion induced renal injury, and it was accompanied by elevated MIF levels and macrophages infiltration. In cultured tubular epithelial cells (TECs), aristolochic acid (AA) promoted matrix production and increased MIF expression, as well as the release of macrophage-related factors. Inhibition of MIF with an antagonist ISO-1 resulted in the abolishment of these genotypes in AA-treated TECs.* Conclusion*. MIF plays an important role in macrophage-related inflammation and matrix deposition in kidney tissues following injury. MIF as a specific inhibitor may have therapeutic potential for patients with inflammatory and fibrotic kidney diseases.

## 1. Introduction

Renal tubulointerstitial fibrosis is a common pathway for all kinds of progressive chronic kidney diseases [1, 2]. Irrespective of the initial cause, renal fibrosis is a dynamic and converging process, which is pathologically characterized by extracellular matrix deposition with inflammatory cell infiltration, tubular epithelial cell loss, and fibroblast accumulation [3]. Excessive and uncontrolled inflammatory responses at the early stages of kidney injury are considered important factors contributing to matrix deposition and fibrosis. Research in recent years has suggested that there is a strong correlation between the infiltration of inflammatory cells, including monocytes and macrophages, and the extent of fibrosis [4].

As the main inflammatory infiltration cells, macrophages in kidney tissues are present in many primary and secondary kidney diseases. Activated macrophages during an inflammatory reaction release a variety of cytokines, chemokines, and bioactive mediators. Increasing evidence suggests that these factors, such as transforming growth factor-*β*1 (TGF-*β*1) [5] and platelet-derived growth factor (PDGF) [6], have profibrogenic effects. They can directly activate fibroblasts and also regulate matrix production by maintaining the balance of various matrix metalloproteinases and tissue inhibitors of matrix metalloproteinases [7, 8]. In addition, these factors can also recruit other inflammatory cells and fibroblasts to exert pro- and antifibrotic effects [9, 10]. It is therefore necessary to investigate the role of macrophage and its regulator factors in inflammatory reaction and matrix deposition in kidney tissues after injury.

Macrophage migration inhibitory factor (MIF), a cytokine discovered in 1966, is recognized as an important immunoregulatory molecule that arrests random immune cell movement [11]. MIF participates in the immune and inflammatory responses of many tissues and organs. In patients with idiopathic pulmonary fibrosis, MIF levels were observed to be significantly higher than the controls. This suggests that MIF is a pleiotropic cytokine involved in the pathogenesis of idiopathic pulmonary fibrosis [12]. In patients with chronic kidney diseases, serum MIF levels were significantly elevated and were associated with markers of oxidative stress and endothelial activation [13]. Therefore, as a critical macrophage regulator, MIF is considered to be an important inflammatory factor involved in the synthesis and accumulation of matrix and renal fibrosis.

Although most studies report that MIF has promotion on inflammation and matrix deposition [12, 14], a recent study suggests that MIF displays a reduced fibrogenic response [15]. In that study, mice with genetically deleted MIF showed strong increases in matrix deposition and fibrosis in two models of chronic liver injury, and MIF markedly inhibited PDGF-induced migration and proliferation of hepatic stellate cells, which were mediated by CD74. These results suggest that MIF exerts inhibitory effects on matrix deposition and fibrosis in the liver. Because of these conflicting effects of MIF on fibrosis, a further study is required for clarification.

To elucidate the role of MIF and the underlying molecular mechanisms involved in matrix deposition and renal fibrosis, this study examined the expression of MIF in kidneys of rats with ureteral obstruction and recanalization. Additionally, MIF expression was analyzed in kidneys with ischemia-reperfusion injury (IRI). In cultured tubular epithelial cells (TECs), the role of MIF was also evaluated during epithelial-to-mesenchymal transition and accumulation of matrix components induced by aristolochic acid (AA). Moreover, a small molecular inhibitor ISO-1 was used to investigate whether inhibition of MIF has a protective effect on inflammation and matrix deposition. Our results suggest that elevated MIF levels* in vivo* and* in vitro* are involved in inflammation and matrix deposition in kidney tissues, and MIF inhibitor may have a therapeutic potential for inflammatory and fibrotic kidney disease.

## 2. Materials and Methods

### 2.1. Animal Model

Thirty-six male Sprague-Dawley rats weighing 180–200 g, 6–8 weeks old, were purchased from the Experimental Animal Center of Wenzhou Medical University (Wenzhou, China). Rats were housed in a temperature-, humidity-, and light-controlled environment and fed a standard rat chow and water. Rats were fasted on the day prior to experiments being conducted. Weight-matched rats were randomly assigned to three groups: (1) an obstruction group, (2) a recanalization group, and (3) an IRI group. These groups were further divided into subgroups, each containing 6 rats. For the obstruction group, the subgroups were sham operation (for 7 days) and UUO (unilateral ureteral obstruction for 7 days). For the recanalization group, the subgroups were BUO (bilateral ureteral obstruction for 1 day) and RBUO (BUO for 1 day and then recanalization for 7 days). For the IRI group, the subgroups were sham (for 24 h) and IRI (ischemia for 45 min and then reperfusion for 24 h). UUO, BUO, and IRI surgery were performed as previously described [16].

The animal study protocols were approved by the Institutional Animal Care and Use Committee of Wenzhou Medical University, China.

### 2.2. Histopathological Examination

Kidney specimens fixed in formalin and embedded in paraffin were cut into 4 *μ*m sections and stained with periodic acid-Schiff (PAS, Yuanye Biotechnology, Shanghai, China), Hematoxylin-Eosin (HE, Yuanye Biotechnology), and Masson's trichrome (Yuanye Biotechnology). Slides were examined and pictures taken using a DM4000 B LED microscope system (Leica Microsystems, Germany) and a DFC 420C 5M digital microscope camera (Leica Microsystems). Tubulointerstitial damage and the degree of interstitial collagen deposition were graded as described previously [17].

### 2.3. Immunohistochemical Staining

Immunohistochemical analysis was performed with 4 *μ*m thick kidney sections that had been dewaxed with xylene and hydrated using sequential ethanol (100, 95, 85, and 75%) and distilled water. Endogenous peroxidase was blocked by 3% hydrogen peroxide. Antigen retrieval was performed by heating sections in 0.1% sodium citrate buffer (pH 6.0). Immunohistochemical staining was performed using the following primary antibodies: type III collagen (1 : 800, Biogot Technology, Shanghai, China), MIF (1 : 1000, Santa Cruz, CA, USA), CD68 (1 : 1000, Santa Cruz), and CD74 (1 : 800, Biogot Technology). Integrated optical density (IOD) value was measured by image analysis. All samples were semiquantitatively or quantitatively assessed by two independent investigators in a blinded manner.

### 2.4. Cell Treatment

A normal rat kidney tubule epithelium (NRK-52E) cell line was obtained from the Cell Bank of Chinese Academy of Sciences (Shanghai, China). NRK-52E cells were maintained in Dulbecco's Modified Eagle Medium (Invitrogen, CA, USA) supplemented with 5% fetal bovine serum (FBS, Invitrogen), 100 U/mL penicillin, and 100 *μ*g/mL streptomycin (Invitrogen). The NRK-52E cells were seeded on six-well culture plates to approximately 70% confluence in the complete medium containing 5% FBS for 24 h and then changed to serum-free medium for 24 h before the treatment with 10 *μ*g/mL aristolochic acid I (lot number A5512, Sigma-Aldrich, St. Louis, MO, USA) with or without 50 *μ*mol/L ISO-1 (lot number D00151457, Merck Chemicals, Darmstadt, Germany).

### 2.5. Immunofluorescence Staining

NRK-52E cells were cultured with AA for 24 h in six-well plates containing glass slides and were then washed with PBS and fixed with 4% paraformaldehyde (Sigma-Aldrich) at 4°C for 30 min. After permeabilization with 0.1% Triton X-100 for 10 min, the specimens were washed with PBS and then blocked with 10% FBS to eliminate the nonspecific fluorescence. Immunofluorescence staining was performed using anti-type III collagen (1 : 800, Biogot Technology), *α*-SMA (1 : 1000, Santa Cruz), E-cadherin (1 : 1000, Abcam, Cambridge, MA, USA), MCP-1 (1 : 800, Biogot Technology), M-CSF (1 : 800, Biogot Technology), or MIF (1 : 1000, Santa Cruz) as the primary antibody, and the cell preparations were incubated with DyLight 488/594 labeled secondary antibodies. The immunocytochemical samples were semiquantitatively or quantitatively assessed by two independent investigators in a blinded manner.

### 2.6. RNA Isolation and PCR Analysis

Total RNA was extracted from rat kidneys or NRK-52E cells using TRIzol reagent (Invitrogen), reverse-transcribed to cDNA templates using a ReverTra Ace qPCR RT kit (Toyobo, Japan). Quantitative RT-PCR (qRT-PCR) was performed using a SYBR Green Real-Time PCR Master Mix Plus (Toyobo). Quality was analyzed on agarose gels, and quantities were measured using Varioskan Flash (Thermo Fisher Scientific, USA). Sequence-specific primers of Col1*α*1, Col3*α*1, ZO-1, E-cadherin, BMP-7, MIF, and *α*-SMA, all listed in Table 1, were synthesized by Invitrogen, and *β*-actin was used as an endogenous reference gene. Samples were analyzed in triplicate. The melting curve was examined to verify that a single product was amplified. For quantitative analysis, all samples were analyzed using the ΔΔCT value method.

### 2.7. Enzyme-Linked Immunosorbent Assay (ELISA)

Rat kidney tissues or cell suspensions were homogenized and centrifuged and the supernatant was collected. Avidin-biotin complex-ELISA was used according to the manufacturer's protocol to determine TGF-*β*1 and MIF levels. ELISA kits were purchased from Xitang Biotechnology (Shanghai, China). All experiments were repeated at least three times.

### 2.8. Western Blot Analysis

Whole proteins from rat kidneys were collected and protein concentrations were determined using a bicinchoninic acid protein assay kit (Beyotime). Whole proteins (20 *μ*g) from each sample were separated by SDS-PAGE and transferred to a polyvinylidene difluoride membrane (Solarbio, Beijing, China). After treatment with 5% skim milk at 4°C overnight, membranes were incubated with various antibodies for 1 h and then incubated with the appropriate horseradish peroxidase-conjugated secondary antibody (Beyotime). Bound antibodies were visualized using chemiluminescence detection on autoradiographic film. The primary antibodies included type III collagen (1 : 100, Biogot Technology), *α*-SMA (1 : 200, Santa Cruz), E-cadherin (1 : 200, Abcam), TGF-*β*1 (1 : 100, Biogot Technology), and MIF (1 : 200, Santa Cruz). Quantification was performed by measuring the intensity of signals using Image-Pro Plus (version 6.0) and normalized to that for the GAPDH antibody (Cell Signaling Technology, MA, USA).

### 2.9. Statistical Analysis

Data are presented as mean ± standard error of the mean. All statistical analyses were performed using the Statistical Package for Social Sciences (version 16.0, SPSS Inc., Chicago, USA). Two-sided Student's *t*-test was used to analyze differences between the two groups. One-way analysis of variance was used when more than two groups were present. A *P* value of <0.05 was considered statistically significant.

## 3. Results

### 3.1. Ureter Obstruction Induced Inflammatory Cell Infiltration, Matrix Deposition, and MIF Expression in Kidneys

**Figure 1:**
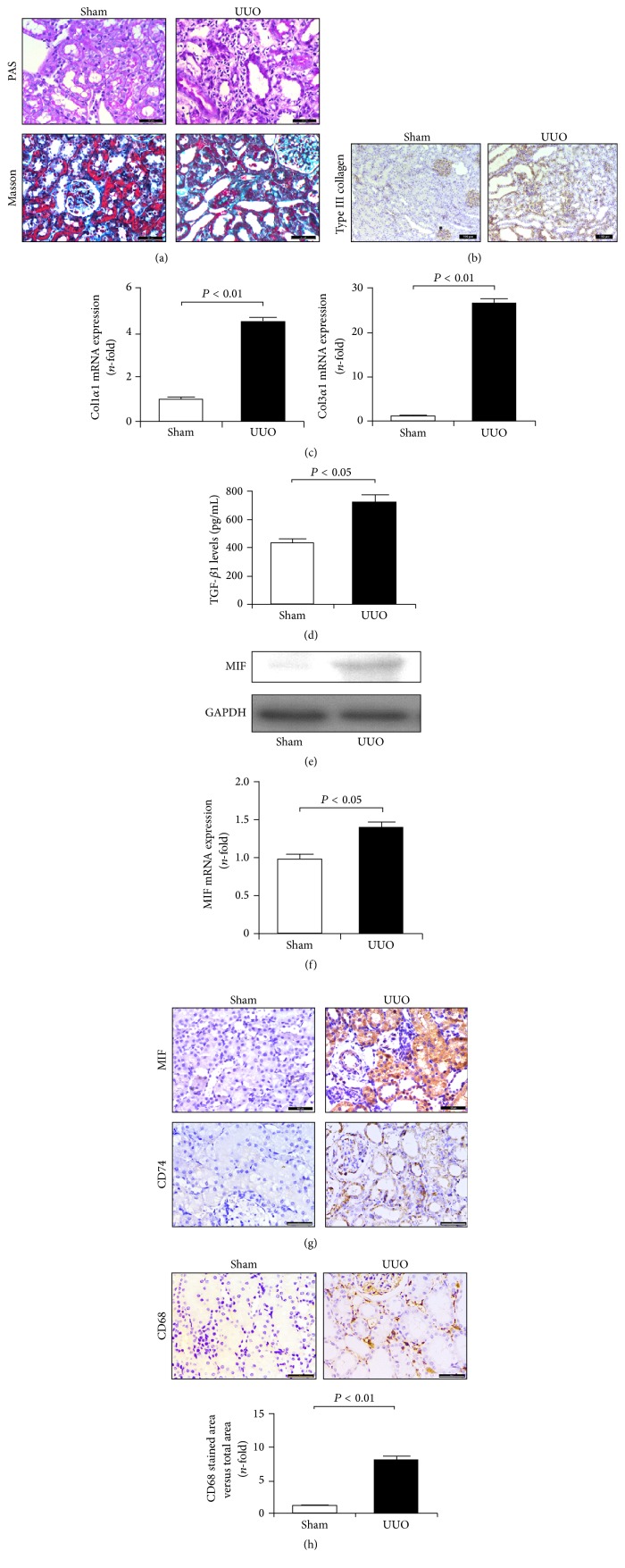
Obstruction-induced matrix deposition and MIF expression in kidneys. (a) PAS and Masson's trichrome staining of renal cortex sections revealed marked tubular dilation and atrophy associated with excessive matrix deposition and inflammatory cells infiltration. Bar: 50 *μ*m. (b) Protein expression of type III collagen determined by immunohistochemical staining was upregulated in UUO kidneys compared with the sham. Bar: 100 *μ*m. (c) qRT-PCR analysis indicated increased mRNA expressions of Col1*α*1 and Col3*α*1 in UUO kidneys. (d) ELISA assay showed enhanced TGF-*β*1 levels in UUO kidneys. (e) Western blot analysis showed increased MIF expression in UUO kidneys. (f) qRT-PCR revealed elevated mRNA expression of MIF in UUO kidneys. (g) Immunohistochemical staining identified upregulated expression of MIF and CD74 in UUO kidneys. Bar: 50 *μ*m. (h) Enhanced CD68 expression reveals marked macrophage infiltration in UUO kidneys. Bar: 50 *μ*m.

Rodent UUO is a well-characterized experimental model resulting in matrix deposition and interstitial fibrosis in kidney tissues [18]. In the obstructed kidneys, PAS staining revealed obvious diffuse congestion and edema and focal hemorrhaging in the renal interstitium, simultaneously accompanied by inflammatory cell infiltration, epithelial cell necrosis, and marked tubular dilation (Figure 1(a)). Additionally, the deposition of total collagen as determined by Masson's trichrome staining showed increased aggravation in UUO rats (Figure 1(a)). These fibrotic changes in the cortical interstitium were confirmed by elevated protein levels of type III collagen (Figure 1(b)) and mRNA levels of Col1*α*1 and Col3*α*1 (Figure 1(c)). Our results also revealed that excessive matrix deposition in the obstructed kidneys was associated with enhanced expression of TGF-*β*1 (Figure 1(d)). Thus, ureteral obstruction promoted TGF-*β*1 expression and matrix accumulation in kidney tissues.

Abnormal MIF expression is regarded as an important process in inflammatory reaction [12, 14, 15]. Here, results from western blot and immunohistochemical staining suggested that MIF levels in UUO kidneys were increased (Figures 1(e) and 1(g)). Similarly, changes in gene levels of MIF as determined by qRT-PCR are consistent with protein levels (Figure 1(f)). Previous study showed that the effect of MIF on macrophages is mediated by its receptor CD74 [15]. Our study showed that CD74 levels in kidneys were markedly increased after an obstruction operation (Figure 1(g)). In addition, high levels of MIF were accompanied with an increasing number of CD68-positive macrophages (Figure 1(h)). These results suggest that MIF is involved in macrophages-mediated inflammation and matrix production.

### 3.2. Recanalization Reduced Inflammatory Cell Infiltration, Matrix Deposition, and MIF Expression in BUO Kidneys

**Figure 2:**
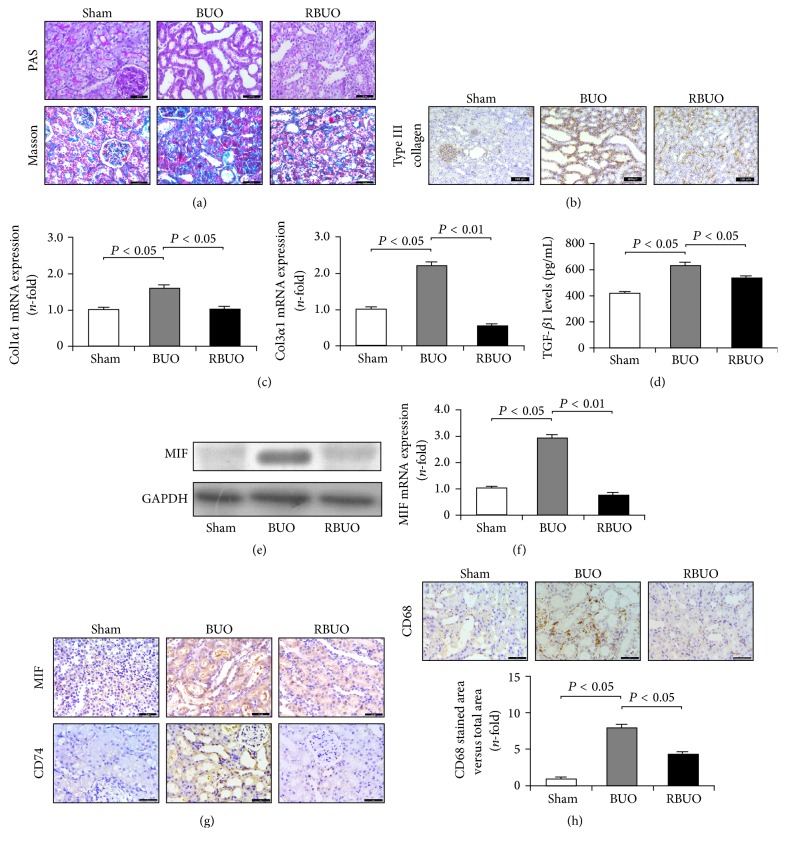
Recanalization reduced matrix deposition and MIF expression in obstructed kidneys. (a) PAS and Masson staining revealed that recanalization operation reduced obstruction-induced kidney injury, matrix deposition, and inflammatory cells infiltration in BUO rats. Bar: 50 *μ*m. (b) Immunohistochemical staining showed that recanalization downregulated obstruction-induced protein expression of type III collagen. Bar: 100 *μ*m. (c) Gene expression levels of Col1*α*1 and Col3*α*1 in BUO kidneys were significantly decreased after recanalization. (d) ELISA assay indicated reduced TGF-*β*1 levels in recanalization kidneys compared with the BUO group. (e) Western blot analysis showed decreased MIF expression in recanalization kidneys. (f) qRT-PCR analysis showed reduced MIF expression in recanalization kidneys compared with the BUO group. (g) Immunohistochemical staining identified downregulated expression of MIF and CD74 in kidneys of recanalization rats. Bar: 50 *μ*m. (h) Recanalization inhibited the infiltration of CD68-positive macrophages in BUO kidneys. Bar: 50 *μ*m.

As mentioned above, upregulated MIF expression is involved in matrix deposition and fibrogenesis. However, whether downregulated expression of MIF also plays an important role in the recovery of fibrosis remains unknown. Thus, a recanalization operation was performed to evaluate this hypothesis. First, we examined the effect of recanalization on matrix deposition and kidney injury in BUO rats. As with the UUO rats, as shown in Figure 2(a), PAS staining revealed that obvious diffuse congestion and edema, epithelial cell necrosis, and marked tubular dilation in BUO kidneys were alleviated after recanalization, simultaneously accompanied by the reduction of infiltrated inflammatory cells. Also, recanalization reduced interstitial fibrosis by suppressing collagen deposition as indicated by Masson staining and reducing the levels of type III collagen by immunohistochemical staining (Figures 2(a) and 2(b)). In addition, decreased expression levels of Col1*α*1 and Col3*α*1 mRNAs were also observed after the recanalization operation (Figure 2(c)). Furthermore, recanalization downregulated the TGF-*β*1 levels in BUO kidneys (Figure 2(d)). These results suggest that the relief from ureteral obstruction by recanalization suppresses effectively TGF-*β*1 expression and matrix production.

In BUO kidneys, inflammatory cell infiltration and excessive matrix deposition were relieved by a recanalization operation. These physiology changes are associated with downregulation in the levels of MIF. As shown in Figures 2(e)-2(g), mRNA and protein expression of MIF in RBUO kidneys showed a marked decrease compared with that in BUO kidneys. The changes in MIF levels were also confirmed by immunohistochemical analysis. In addition, the expression of CD74 in RBUO kidneys was significantly decreased, accompanied by a downregulated number of CD68-positive macrophages (Figures 2(g) and 2(h)). Thus, these results revealed that CD74-mediated downexpression of MIF is involved in the recovery of renal fibrosis.

### 3.3. Inflammatory Cell Infiltration and Overexpression of MIF Occurred in IRI Kidneys

**Figure 3:**
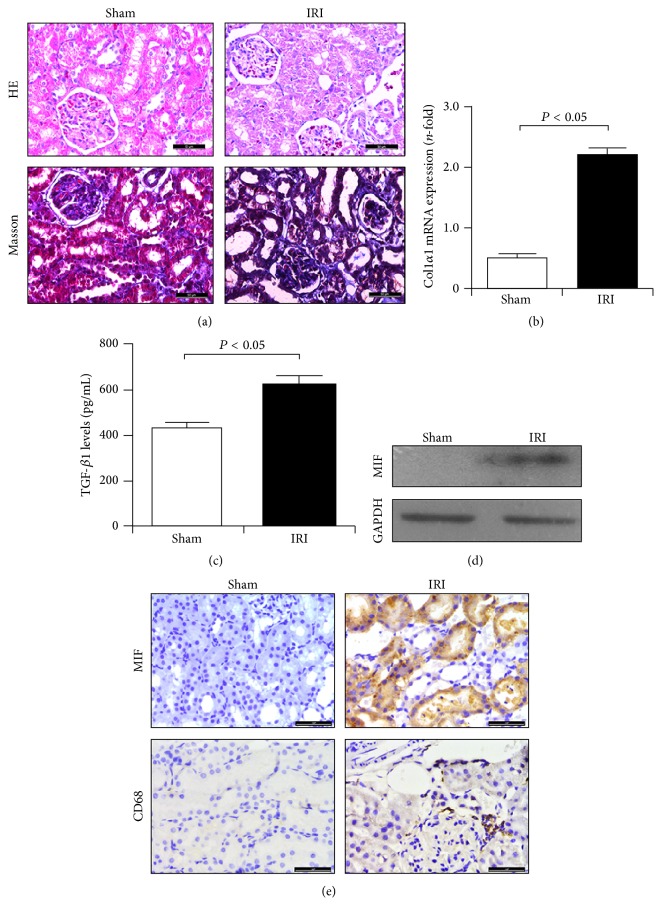
Matrix deposition and MIF expression were enhanced in IRI kidneys. (a) HE and Masson staining revealed marked inflammatory cell infiltration and interstitial injury in IRI kidneys tissues of BUO rats. Bar: 50 *μ*m. (b) Gene expression of type III collagen in IRI kidneys was upregulated. (c) IRI induced the upregulation of TGF-*β*1 levels in IRI kidneys. (d) IRI induced upregulated expression of MIF in IRI kidneys. (e) Increased expression of MIF and CD68 in kidneys of IRI rats. Bar: 50 *μ*m.

Given that MIF plays an important role in renal inflammation and fibrogenesis, we considered whether similar results can reoccur in kidneys following ischemia-reperfusion injury. Thus, in this study, the infiltration of macrophages and the expression of MIF and CD74 in IRI kidneys were evaluated. As shown in Figure 3(a), HE and Masson staining revealed marked tubulointerstitial injury and increasing collagen's synthesis. Also, the gene expression of type I collagen (Col1*α*1) was significantly enhanced (Figure 3(b)), indicating matrix deposition in IRI kidneys. These changes in kidneys may be a result of increasing TGF-*β*1 levels (Figure 3(c)). In addition to these fibrotic changes, IRI also induced excessive deposition of CD68-positive macrophages and MIF expression (Figures 3(d) and 3(e)), and enhanced MIF expression was closely related to macrophage infiltration. Thus, these findings reconfirmed the key role of MIF in macrophage-related inflammatory reaction and matrix deposition.

### 3.4. AA Induced EMT, Matrix Accumulation, and MIF Expression in TECs

**Figure 4:**
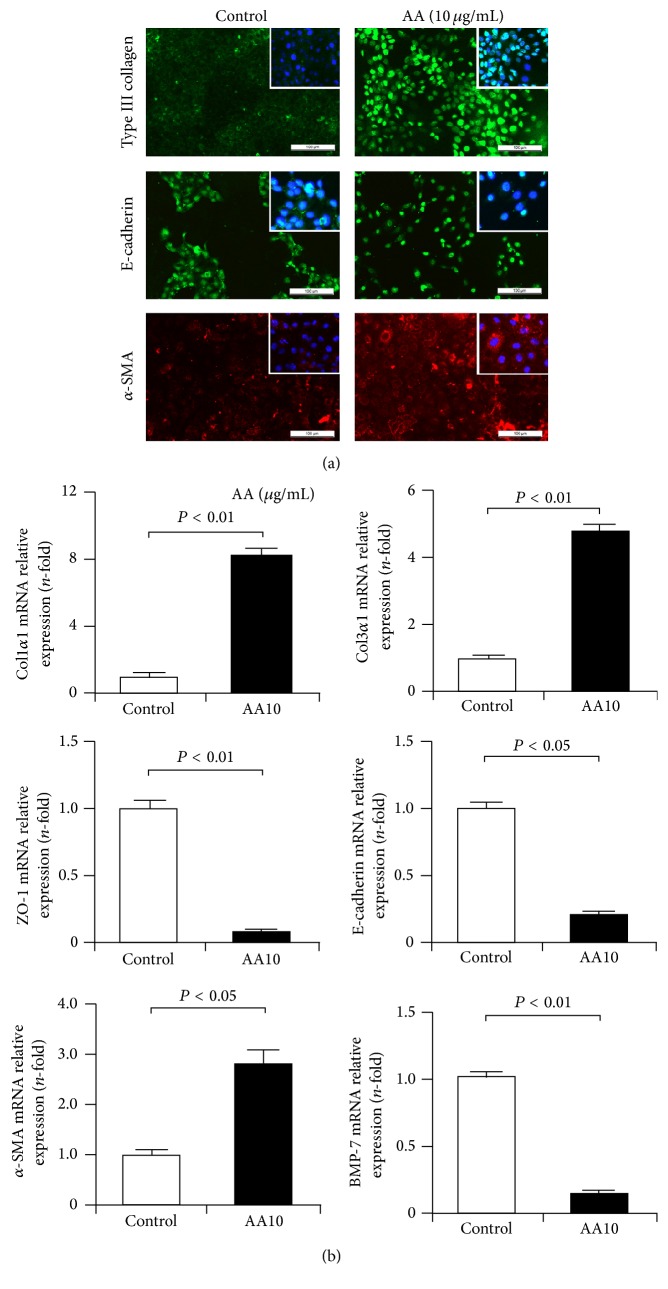
EMT and matrix deposition in AA-treated TECs. (a) Immunofluorescence staining revealed enhanced expression of type III collagen and *α*-SMA and decreased expression of E-cadherin in AA-treated NRK-52E cells. Bar: 100 *μ*m. (b) qRT-PCR assay showed that the mRNA expression of Col1*α*1, Col3*α*1, and *α*-SMA was upregulated in NRK-52E cells after AA treatment, and the expression of ZO-1, E-cadherin, and BMP-7 was downregulated.

Firstly, the expression levels of matrix components type I and III collagens were examined in cultured NRK-52E cells after AA injury. We found that AA upregulated the protein expression of type III collagen as indicated by immunofluorescence staining (Figure 4(a)) and mRNA expression of Col1*α*1 and Col3*α*1 as demonstrated by qRT-PCR (Figure 4(b)). As a key process in fibrogenesis, the EMT process was also evaluated in AA-treated NRK-52E cells. Immunofluorescence staining revealed that AA decreased the expression of the epithelial marker E-cadherin protein and increased expression of the mesenchymal marker *α*-SMA (Figure 4(a)). In addition, downregulated expression of E-cadherin and ZO-1 and upregulated expression of *α*-SMA mRNAs were also observed in AA-treated cells (Figure 4(b)). Moreover, AA reduced the mRNA expression of the EMT inhibitor BMP-7 (Figure 4(b)). These findings suggest that AA induced fibrotic phenotypes in NRK-52E cells.

**Figure 5:**
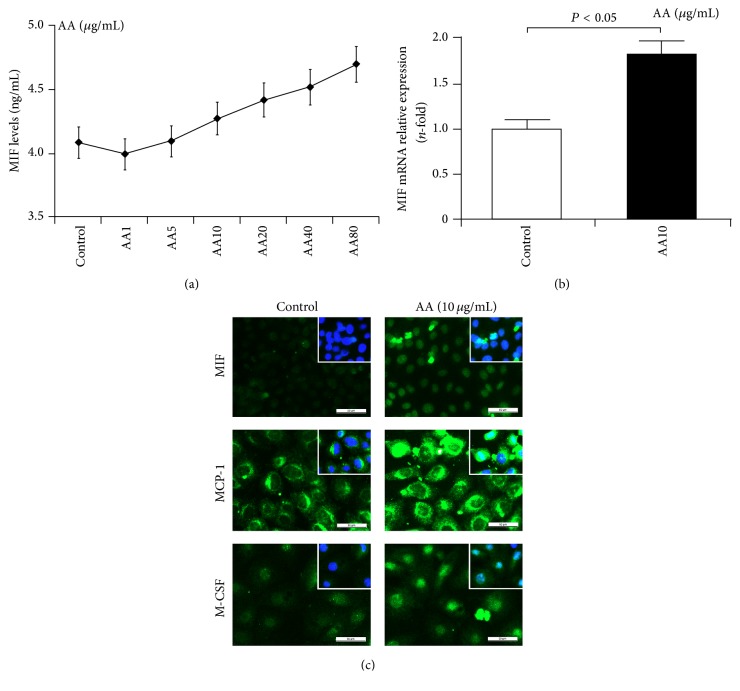
Enhanced MIF expression in TECs after AA injury. (a) ELISA assay revealed AA increased MIF levels in NRK-52E cells in a concentration-dependent manner. (b) qRT-PCR assay showed that MIF mRNA expression was increased in AA-treated NRK-52E cells. (c) Immunofluorescence staining revealed enhanced expression of MIF, M-CSF, and MCP-1 in AA-treated NRK-52E cells. Bar: 50 *μ*m.

Secondly, we examined the synthesis and release of MIF in NRK-52E cells after AA injury for 24 h. Results from ELISA showed that AA increased MIF levels in an obvious concentration-dependent manner (Figure 5(a)). In addition, immunofluorescence staining and qRT-PCR also revealed that the expression of MIF is upregulated in NRK-52E cells after AA injury (Figures 5(b) and 5(c)). In addition to MIF, AA also increased the expression of monocyte chemoattractant protein-1 (MCP-1/CCL2) and macrophage colony stimulating factor (M-CSF) (Figure 5(c)). These results suggest that AA induces an inflammatory response and MIF-related macrophages might be involved in this process and matrix deposition.

### 3.5. Inhibition of MIF Abolished EMT Induction and Matrix Deposition in AA-Treated TECs

**Figure 6:**
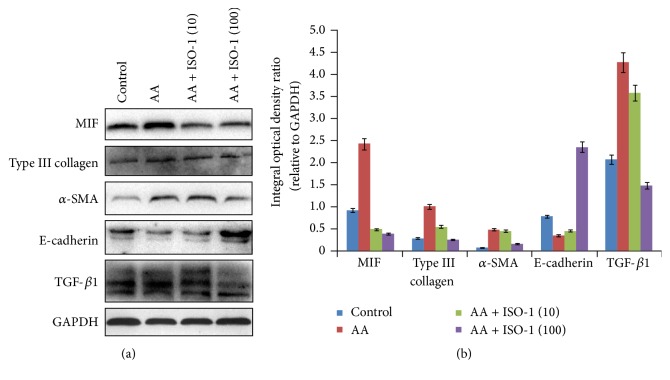
Inhibition of MIF abolished AA-induced EMT induction and matrix deposition in TECs. Western blot assay showed that AA increased the expression of MIF, type III collagen, *α*-SMA, and TGF-*β*1 and decreased the expression of E-cadherin. However, these changes including EMT induction and matrix deposition were inhibited by ISO-1 treatment.

As mentioned above, enhanced MIF expression is positively associated with AA-induced EMT induction and matrix deposition in TECs, but it is unknown whether downregulated expression of MIF may abolish AA-induced fibrotic phenotypes. To test this hypothesis, in this study, a MIF antagonist ISO-1 was also used in cultured NRK-52E cells. We found that ISO-1 significantly decreased the levels of MIF in TECs after AA treatment (Figure 6). In addition, AA-induced upregulation in *α*-SMA expression and downregulation in E-cadherin expression were reversed after ISO-1 treatment. Furthermore, ISO-1 also reduced the expression of type III collagen and the levels of TGF-*β*1. Thus, inhibition of MIF with ISO-1 resulted in the abolishment of the induction of EMT and deposition of matrix in NRK-52E cells after injury.

## 4. Discussion

In this study, we first identified obvious renal injury and tubulointerstitial fibrosis in the kidney tissues of UUO rats. Additionally, elevated expression of MIF and its receptor CD74 and upregulated numbers of CD68-positive macrophages were observed in the obstructed kidneys. Relief of obstruction-induced fibrosis by a recanalization operation decreased the levels of MIF and CD74 and downregulated CD68-positive macrophage numbers. Similarly, in IRI rats, excessive matrix deposition and increased macrophage accumulation were accompanied with upregulated synthesis and release of MIF. These findings indicated that MIF plays an important role in the infiltration and activation of macrophages, excessive matrix accumulation, and fibrogenesis in kidney tissues.

Renal fibrosis is proposed to be an orchestrated, highly regulated process that consists of inflammation, matrix deposition, and scar formation [19]. Inflammatory reactions and moderate matrix deposition are known to be a reversible phase, and inhibition of an inflammatory reaction and decreased secretion of chemokines and proinflammatory cytokine may be an effective therapeutic strategy for renal fibrosis [19, 20]. As the main nucleated cells in kidney tissues, macrophages are identified as critical regulators of inflammation during fibrosis [4]. Macrophages can express many cytotoxic moieties, including proteolytic enzymes, proinflammatory cytokines, and chemokines [21]. The functions of macrophages may be partially regulated by MIF [22]. Thus, it is important to investigate the role of MIF and its association with macrophages in inflammation and matrix deposition.

MIF is a key molecule in macrophage migration and accumulation at sites of injury and is involved in many inflammatory reactions during fibrogenesis, such as glomerulonephritis and lupus nephritis [22–24]. In the rat model of crescentic antiglomerular basement membrane glomerulonephritis, enhanced MIF expression by intrinsic cells contributes to macrophage accumulation and severe tissue damage, including crescent formation [23]. Increased MIF expression was also observed in MRL/lpr mice, and MIF deficiency attenuates macrophage recruitment, glomerulonephritis, and lethality in MRL/lpr mice [22]. In addition, a transgene of MIF induces podocyte injury and progressive mesangial sclerosis in the mouse kidney [25]. Thus, these findings indicated that MIF has proinflammatory and profibrotic effect in renal tissues following injury. However, surprisingly, a study by Rice et al. showed that the progression of renal injury in obstructive nephropathy is independent of MIF by using MIF-knockout mice. It seems that MIF may negatively contribute to an inflammatory reaction and renal fibrosis. This puzzling role of MIF, we hypothesized, may correlate to the physiological function of macrophages after injury.

Recent studies on macrophages biology and differentiation have revealed their pleiotropic activities [26]. Macrophages can be divided into M1 and M2 subpopulations. In the development and recovery of renal diseases, these subpopulations may exert their functionality independently [27]. M1 macrophages have a pathogenic function in renal inflammation, making them a logical target for elimination. Alternatively, M2 macrophages resolve inflammation and repair injury, making them a potential therapeutic tool against renal injury. Thus, at different time points after injury, the role of MIF in an inflammatory reaction and renal matrix deposition may be associated with the polarization of macrophages. In the early period of injury, MIF induces the transition of macrophages to M1 subpopulation and thereby mediates the inflammation and promotes matrix deposition. In the later period, MIF-mediated M1 macrophages are at a disadvantage and thereby promote recovery. Inhibition of MIF expression to accelerate the recovery of disease including inflammation and matrix deposition may be promising.

Previous studies have reported that MIF is expressed in various kinds of cells, including mononuclear macrophages, epithelial cells, and smooth muscle cells [28–30]. In the present study, we found that MIF is abundantly expressed in cortical tissue, especially at the basal aspect of epithelial cells around renal tubules. Epithelial cells are a target cell for MIF that has been confirmed in the* in vitro* experiments. Epithelial cells are immunologically active resident cells that can interact with other immune effector cells [31, 32]. MIF was also detected in some glomeruli and the medulla, but expression was very weak, findings that are similar to other models of inflammatory renal disease [22]. In the present study, the activity of MIF in NRK-52E cells was significantly increased in AA-induced EMT and matrix deposition. Although in the* in vitro* experiment there were no macrophages, overexpression of MIF as well as other macrophage-related factors including MCP-1 and M-CSF suggested the possibility that macrophages were induced and activated* in vivo*. AA-mediated MIF expression was in a concentration-dependent manner, indicating that the release of macrophage-related inflammatory factors may be associated with the extent of injury, including EMT induction and matrix deposition. Inhibition of MIF activity with a small antagonist ISO-1 not only resulted in the reduction of EMT and matrix deposition, but also decreased TGF-*β*1 levels. Thus, these findings reconfirmed the important role of MIF in macrophage-mediated inflammation, matrix deposition, and renal fibrosis.

It should be noted that RBUO rats were used for the recanalization model rather than RUUO rats (recanalization after UUO) for the following reasons: (i) tubulointerstitial lesions and matrix deposition are not obvious in UUO after 1 day, so it is unlikely that recanalization would be of much significance, and (ii) if UUO time is more than 1 day, ureteral damage is severe, and it is not suitable for recanalization, although tubulointerstitial lesions and matrix deposition are obvious.

In conclusion, our* in vivo* and* in vitro* experiment identified the crucial role of MIF in macrophage-related inflammation, matrix deposition, and renal fibrogenesis. MIF as a specific inhibitor may have therapeutic potential for patients with inflammatory and fibrotic kidney diseases.

## Figures and Tables

**Table 1:** qRT-PCR primers in this study.

Gene	Sequence (5′→3′)	GenBank accession	Product size (bp)
Col1*α*1	GATCCTGCCGATGTCGCTAT (F)	NM_053304.1	276
	GGAGGTCTTGGTGGTTTTGTATTC (R)		

Col3*α*1	AAGGCTGAAGGAAATAG (F)	NM_032085.1	147
	AATGTCATAGGGTGCGATA (R)		

ZO-1	AACAGAGCCGAGCAGTTAGCC (F)	NM_001106266.1	238
	CAACATCAGCAATCGGTCCA (R)		

E-cadherin	GTGCCACCACCAAAGATA (F)	NM_031334.1	195
	GGCTGAGACAACCCTAAT (R)		

*α*-SMA	GGCATCCACGAAACCACCT (F)	NM_031004.2	212
	CCGCCGATCCAGACAGAAT (R)		

BMP-7	GTGGTCAACCCTCGGCACA (F)	NM_001191856.1	215
	GGCGTCTTGGAGCGATTCTG (R)		

MIF	TCTCCGCCACCATGCCTATG (F)	NM_031051.1	178
	GGGTCGCTCGTGCCACTAAA (R)		

*β*-actin	CCCATCTATGAGGGTTACGC (F)	NM_031144.2	150
	TTTAATGTCACGCACGATTTC (R)		
